# Myospasm and Fasciculation After Botulinum Toxin Injection

**DOI:** 10.1093/asjof/ojaf027

**Published:** 2025-04-24

**Authors:** Xueqing Li, Zhaowei Zhu, Min Zhu, Yanting Zheng

## Abstract

Botulinum toxin type A (BTA) is commonly used to treat dystonia; however, reports of dystonia caused by BTA are rare. In this case study, the authors aimed to illustrate the instances of dystonia after cosmetic injections of BTA. Three cases were retrospectively analyzed, with follow-up evaluations conducted to assess the development and resolution of symptoms. Injection techniques, including microdroplet and intradermal methods, were examined, and potential hypotheses regarding the etiology of dystonia were explored. Recommendations for mitigating adverse effects were formulated based on the analysis of injection practices, including modifications to dosage, injection-site distribution, and volume. In all cases, patients experienced transient dystonia following BTA injections, with symptoms including muscle spasms and abnormal muscle contractions, which resolved spontaneously or with minor interventions. The findings suggest that the use of microdroplet techniques and careful injection strategies can minimize the risk of such adverse effects. In conclusion, the authors suggest that neurological injury, neuromuscular junction impact, uneven drug distribution, and imbalance between denervation and functional recovery may cause dystonia.

**Level of Evidence: 5 (Diagnostic):**

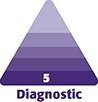

The use of botulinum toxin has been increasing, with new indications and applications continually emerging, along with reports of adverse reactions.^1,2^ Botulinum toxin type A (BTA), one of the most commonly used forms of botulinum toxin, has a long history of use for treating dystonia.^3^ However, cases of dystonia resulting from BTA injections are rarely mentioned in randomized controlled trials (RCTs), which often provide limited follow-up and analysis. Because of the widespread use of BTA for cosmetic purposes, both doctors and consumers are increasingly concerned about adverse reactions. In China, all available botulinum toxin products are BTA, with Prosigne (Lanzhou Institute of Biological Products, Gansu) and Botox (Allergan, Irvine, CA) being the most widely used. As a plastic surgeon with 7 years of experience and having administered ∼6000 BTA injections, I have documented 3 cases of postinjection dystonia. These include 1 case of frontalis muscle spasm, 1 of frontalis muscle fasciculation, and 1 of chin muscle spasm and fasciculation. An analysis of the possible causes and recommendations is presented.

## CASE DESCRIPTION

### Case 1

A 32-year-old woman received her first Prosigne injection in the frontal and interbrow lines in August 2024. She had previously received irregular Prosigne and Botox injections in the masseter, platysma, and trapezius muscles. Notably, she had experienced left-sided Bell's palsy twice, 15 and 10 years earlier, resulting in mild facial reanimation, with the left eyebrow being lower and flatter than the right side—sequelae of peripheral facial paralysis—without prosopospasm or other symptoms related to facial muscles.

For the frontalis muscle, injections were administered 1.5 cm above the eyebrow using a 2-line parallel, staggered distribution method with 8 subcutaneous injection sites, delivering 2 U (concentration: 40 U/mL) at each site with a 29 G needle. The glabella complex received 5-point intramuscular injections: 1 U/side in the corrugator supercilii, 6 U/side in the depressor supercilii, and 2 U in the descending interbrow muscle (also at 40 U/mL), using a 29 G needle. Two weeks after the injection, the patient was satisfied with the result.

**Figure 1.**
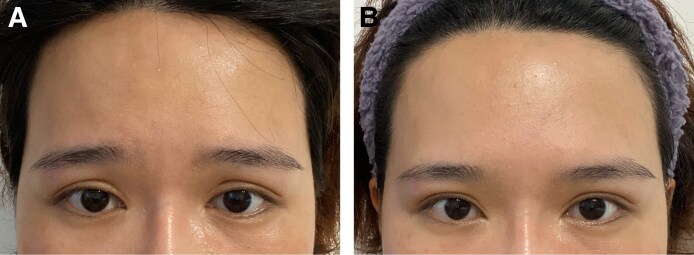
A 32-year-old female received HengLi (Lanzhou Biological Products Institute, Lanzhou, China) in the frontal and interbrow lines: (A) when triggering a spasm at 4 weeks and (B) normal condition at 4 weeks.

However, 3 weeks postinjection, she experienced a tightening sensation in the center of her forehead after inadvertently scratching it. This led to an unusual clustering of her eyebrows, giving her a “hurt and wronged” appearance, and she was unable to control the movement of her eyebrows voluntarily (Figure 1, Videos 1, 2). This phenomenon resolved within 5 minutes after the patient vigorously massaged her forehead. Further examination revealed that pressure massage did not trigger myospasm, although transverse, longitudinal, or zigzag scratch movements did. A wait-and-see approach typically allows myospasms to resolve spontaneously within 5 minutes. By Week 8 postinjection, the intensity of the myoclonus had significantly decreased, and the duration was reduced to 1 minute. By 4 months postinjection, the abnormal muscle spasms had completely resolved. Electromyography (EMG) performed at Week 8 revealed neurogenic damage to the bilateral frontalis muscles. The motor afferents of the zygomatic, buccal, and mandibular marginal branches of the bilateral facial nerves were normal, and the latency and frequency of *F*-wave appearance in the bilateral facial nerves were normal. A large number of abnormal spontaneous potentials were observed in the resting state of the bilateral frontalis EMG. The left side was attributed to sequelae of previous peripheral facial nerve paralysis, whereas the right temporal branch of the facial nerve was considered to be related to injection injury.

### Case 2

A 42-year-old woman received her first Prosigne injection in the frontal area in April 2022, with no previous history of BTA injections elsewhere. The patient maintained her preexisting fitness level.

The frontalis muscle injection was administered following the method described in Case 1. One week postinjection, the patient reported a spontaneous throbbing sensation on the right side of her forehead. When this occurred, the right-side eyebrow was raised and remained fixed. This abnormal phenomenon was alleviated by applying firm pressure to the area for ∼10 s and resolved spontaneously within ∼30 s without intervention. These symptoms occasionally recurred for 3 months postinjection, with an average frequency of 1 to 3 times per day, and ultimately resolved after 3 months.

### Case 3

A 30-year-old woman received her first Prosigne injection in the chin in May 2022. She had previously received Prosigne injections for frown lines and crow's feet. The patient maintained good physical fitness. The chin muscle was injected using a 3-point intramuscular method, forming an equilateral triangle in the mid-chin area, with 2 U (40 U/mL) delivered at each site using a 29 G needle.

**Figure 2.**
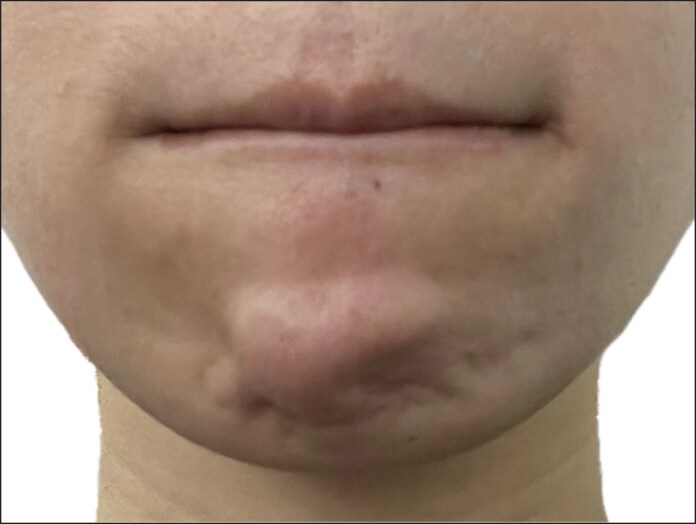
A 30-year-old female received HengLi (Lanzhou Biological Products Institute, Lanzhou, China) in the chin, when triggering a spasm at 1 week.

Three days after the injection, the patient noticed tightness in her chin and experienced abnormal elevation of the chin. This elevation was accompanied by myospasm of the surrounding muscles when pouting or pursing the lips (Figure 2). The myospasm was relieved by self-massage and resolved within 3 minutes without further intervention. Considering the paradoxical bulging of the mentalis, a supplementary injection was suggested; however, the patient refused further treatment. Pouting or lip pursing occasionally triggered similar symptoms within 6 months of injection, with an average frequency of 3 times per day. Six months postinjection, the phenomenon gradually diminished, transitioning to involuntary twitching of the chin, which occurred 1 to 3 times per day until ∼2 years postinjection. Twenty-eight months after the injection, the chin twitching eventually disappeared.

## DISCUSSION

In addition to its cosmetic uses, BTA, by reducing the release of the neurotransmitter acetylcholine, is widely used in the treatment of dystonia, neuralgia, and hyperhidrosis.^4,5^ Regarding aesthetics, RCTs on glabellar lines are relatively common. In 2004, Ascher et al reported 1 (1/34) instance of forehead muscle spasm in the 50 U group using Dysport (Galderma, Lausanne, Switzerland), with other groups receiving 25 U, 75 U, or placebo.^6^ In 2005, they reported 1 (1/50) muscle spasm in the glabellar area vs 0/50 in the placebo group.^7^ Brandt et al, in a study also using Dysport, reported 1 (1/105) case of blepharospasm in the Dysport group, and the placebo group had 2 (2/53) cases of blepharospasm.^8^ Carruthers and Carruthers reported a total of 3 cases, including 2/20 in the 20 U group and 1/20 in the 60 U group, as well as 2 (2/20) instances of brow/lid spasms or muscle contractions when using Botox, which included 4 groups: 20, 40, 60, and 80 U.^9^ Kane et al reported 1 (1/544) case of blepharospasm in the Dysport group compared with 0/272 in the placebo group.^10^ Although these adverse reactions have been documented, their specific characteristics have rarely been described. These reactions were generally considered mild and transient and thus not taken seriously enough; however, the patients reported in this article indicated that the phenomenon was disturbing their daily lives. Although injections in the glabellar complex may cause spasms in the forehead, based on corroborating EMG findings, we believe that the frontal spasms in Case 1 were caused by injections in the frontalis muscle.

After discussions with peers, 1 case of occasional fasciculation was reported to improve days after a masseter injection; this symptom disappeared within 2 weeks without specific treatment. Another colleague reported twitching around 1 eye after mesotherapy with hyaluronic acid–mixed BTA, although there was no follow-up in this case. Paradoxical bulging of the mentalis has been attributed to uneven drug diffusion,^11^ and supplementary injections can resolve this issue; however, there are no reports of chin spasms or twitching.

We propose the following hypotheses regarding the mechanisms underlying these phenomena following a BTA injection.

### Neurological Injury

Cosmetic injections are generally guided by anatomical landmarks, injection angle, depth, and the feel of the injection to determine the correct location. Although blind injections are used, a strong understanding of anatomical structures and extensive experience can help avoid damage to critical blood vessels and nerves. Nonetheless, certain risks remain.^12,13^ In particular, some structures, such as the temporal branch of the facial nerve, are complex in composition, alignment, and hierarchy.^14^ Although needlestick injuries may not typically cause permanent nerve damage, they should not be overlooked.

### Neuromuscular Junction Impact

BTA blocks the release of acetylcholine at the neuromuscular junction, which paralyzes the target muscles. Mechanical stimulation, such as massage, may trigger action potentials within the muscles, leading to contraction.^15^ Discomfort from involuntary muscle contraction may prompt the patient to apply pressure to the area; however, this does not necessarily alleviate the spasm. As observed in Case 1, the release time was essentially the same, regardless of whether massage was applied.

### Uneven Drug Distribution

Injections may not diffuse uniformly, leading to inhibition in certain muscle bundles, whereas unaffected bundles may compensate by increasing stimulus signals.

### Imbalance of Denervation and Functional Recovery

Prolonged episodes of chin muscle throbbing may be linked to an imbalance in terminal sprouting, degeneration of excessive budding, motor endplate metamorphosis, and the functional recovery of the axon.^16^

To avoid the occurrence of dystonia, we believe that it is preferable to adopt the conventional injection method. For patients who develop dystonia after BTA injection, microdroplet and intradermal injections can be used for the treatment of frontal lines, ensuring that the injection sites are more evenly dispersed, thereby minimizing the probability of nerve injury.^17^ When injecting the mentalis, a combination of shallow and deep infiltrations, along with careful consideration of the appropriate dose and volume, can help achieve effective dissipation. Given that the placebo group in RCTs also experienced myoplasm, minimizing unnecessary needle sticks is crucial. If this phenomenon occurs, particularly when triggering factors are present, patients should be advised to avoid triggering movements and consider taking oral methylcobalamin, the active form of Vitamin B12, which acts as a neurotrophic repair agent.

A limitation of this study is that another EMG was not performed on Case 1 after complete resolution of symptoms to determine the relationship between frontal muscle spasm and nerve injury. Cases 2 and 3 did not undergo EMG to detect the presence of neuromuscular lesions. Over the course of 7 years of practice, not all patients were followed up, resulting in the loss of data. To investigate this phenomenon further, we will conduct additional follow-ups and explore the relationship between the occurrence of this phenomenon and factors, such as the type of botulinum toxin, needlestick injuries, injection techniques, and the concentration and volume of the injections.

## CONCLUSIONS

Botulinum toxin injections may cause dystonia, which is generally short lived and reversible. Because of its low incidence, routine injection techniques are considered sufficient. The effects of botulinum toxin, needle injury, and the degree of diffusion of the drug may all contribute to the development of dystonia. A balance of safety and efficacy should be prioritized when administering botulinum toxin therapy.

The study was conducted in compliance with the Declaration of Helsinki. All patients signed an informed consent form and consented to the disclosure of imaging data.

## Supplementary Material

ojaf027_Supplementary_Data
